# Customizing cancer treatment at the nanoscale: a focus on anaplastic thyroid cancer therapy

**DOI:** 10.1186/s12951-023-02094-9

**Published:** 2023-10-13

**Authors:** Jingjing Wang, Jie Tan, Bian Wu, Ruolin Wu, Yanmei Han, Chenyang Wang, Zairong Gao, Dawei Jiang, Xiaotian Xia

**Affiliations:** 1 Department of Nuclear Medicine, Union Hospital, Tongji Medical College, Huazhong University of Science and Technology, No.1277 Jiefang Avenue, 430022 Wuhan, Hubei PR China; 2 Hubei Province Key Laboratory of Molecular Imaging, Wuhan, China; 3 Key Laboratory of Biological Targeted Therapy, the Ministry of Education, Wuhan, China; 4 Department of Breast and Thyroid Surgery, Union Hospital, Tongji Medical College, Huazhong University of Science and Technology, Wuhan, China; 5 Cancer Center, Union Hospital, Tongji Medical College, Huazhong University of Science and Technology, Wuhan, 430022 China

**Keywords:** Anaplastic thyroid cancer, Nanomaterials, Nanomedicine, Therapeutic mechanism, Clinical application

## Abstract

Anaplastic thyroid cancer (ATC) is a rare but highly aggressive kind of thyroid cancer. Various therapeutic methods have been considered for the treatment of ATC, but its prognosis remains poor. With the advent of the nanomedicine era, the use of nanotechnology has been introduced in the treatment of various cancers and has shown great potential and broad prospects in ATC treatment. The current review meticulously describes and summarizes the research progress of various nanomedicine-based therapeutic methods of ATC, including chemotherapy, differentiation therapy, radioiodine therapy, gene therapy, targeted therapy, photothermal therapy, and combination therapy. Furthermore, potential future challenges and opportunities for the currently developed nanomedicines for ATC treatment are discussed. As far as we know, there are few reviews focusing on the nanomedicine of ATC therapy, and it is believed that this review will generate widespread interest from researchers in a variety of fields to further expedite preclinical research and clinical translation of ATC nanomedicines.

## Introduction

Anaplastic thyroid cancer (ATC) is a rare but highly aggressive malignancy composed of undifferentiated follicular thyroid cells that is rapidly fatal in the vast majority of cases [1, 2]. ATC accounts for only 1–2% of all thyroid cancers, but it is responsible for more than 50% of thyroid cancer deaths [3]. The median overall survival of patients with ATC is approximately 3–5 months, and less than 20% of affected patients are still alive 1 year after diagnosis [4, 5]. Clinically, ATC typically affects elderly individuals, with the majority being over 60 years old and predominantly female [2].

ATC is characterized by a rapid onset, causing symptoms such as hoarseness, dysphagia, and dyspnea. Currently, the most commonly employed treatment strategies for ATC include excision surgery, external beam radiation therapy (EBRT), and chemotherapy [6, 7]. However, the majority of patients with ATCs are considered stage IV at diagnosis, as distant metastatic disease is highly probable either at or shortly after diagnosis, making them ineligible for surgery [8, 9]. EBRT ought to be taken into consideration early on in the treatment of ATC. However, EBRT is associated with severe side effects, depending on the radiation dose [10–12]. Chemotherapy is also used to treat ATC. However, multiple drug resistance (MDR) and various adverse drug reactions, such as cardiotoxicity, myelosuppression, and digestive tract injury, make cancer treatment highly prone to failure [13]. One of the new emerging therapeutic approaches in ATC is the application of targeted therapy [14]. Nonetheless, the use of targeted agents still poses challenges due to side effects and the development of tumor cell resistance. Despite various therapeutic strategies for ATC, tumor cells have the capacity to develop resistance to therapy and have shown only limited survival benefits in patients with ATC. Thus, novel therapeutic methods are urgently needed to combat ATC.

**Fig. 1:**
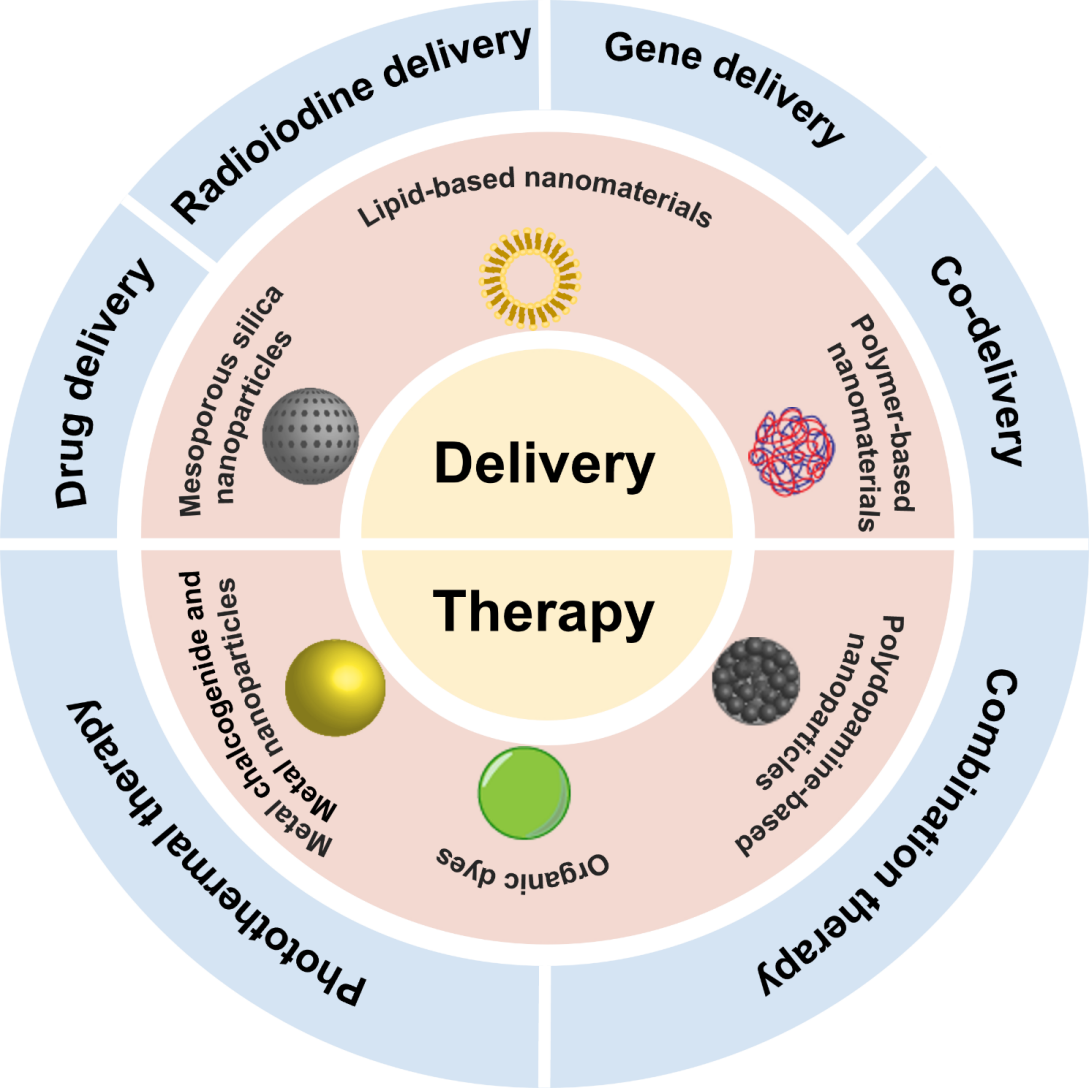
Scheme illustration of nanomedicines in current treatment approaches of ATC.

In the era of precision medicine, nanomaterials have received a lot of attention and have been extensively investigated as promising medical reagents due to their special physicochemical properties, such as controllable shape and size, high permeability, large surface area, and excellent quantum properties [15, 16]. Nanomaterials possess significant advantages in the diagnosis and therapy of tumors [17, 18]. However, there are few studies about ATC nanotechnology-based diagnostic methods, while most studies have focused on therapy [19, 20]. In this review, we discussed the innovative developments of nanomedicine applied to ATC therapy. Nanomaterials have remarkable potential to enhance the efficacy of ATC therapeutics by serving as both drug carriers and therapeutic agents. Nanomaterials can improve the efficacy of chemotherapeutic agents in ATC therapy by enhancing their bioavailability and reducing adverse effects. Selective delivery of differentiation agents by nanomaterials to ATC cells may improve the re-differentiation of ATC. Nanocarriers make it possible to apply radioiodine to ATC therapy via active or passive targeting to deliver radioiodine to the tumor site. By promoting their uptake and accumulation at tumor sites and cells, nanostructures have the potential to enhance the effectiveness of genetic tools in the regulation of gene expression. Furthermore, nanomaterials can provide photothermal therapy for ATC suppression. The nanomaterials have also been developed for combination therapy, providing possible approaches to overcome ATC therapeutic challenges. Despite significant efforts to research and develop nanomedicine in preclinical situations, no nanomedicines have been approved for ATC therapy. The current review provides a detailed description and summary of nanomedicines for the different kinds of ATC therapeutic methods based on lots of pertinent literatures, aiming to find the most promising nanomedicines for therapeutic needs. Furthermore, future challenges and perspectives in this field are also presented to advance the development of nanotherapeutics for ATC management. Figure 1 and table 1 provides a summary of the nanomedicine designed for ATC treatment.

**Table 1:** Overview of nanomedicine designed for treatment in ATC

Treatment strategies	Therapeutic agent	Applied nanoparticles	Key findings	References
Chemotherapeutic drug delivery	Doxorubicin	Mesoporous organosilica nanoparticles	High ratio of drug loading and intracellular accumulation of DOX in drug-resistant ATC cells	[24]
	Doxorubicin	Dopamine-melanin nanoparticles	Increase the internalization of DOX in drug-resistant ATC cells	[30]
	Doxorubicin	Nanobubbles	Increase intracellular drug content and enhance DOX cytotoxicity	[31]
	Doxorubicin	Nanobubbles	Reduce tumor volume and weight, extend tumor doubling time, and diminish the cardiotoxicity of DOX	[32]
	Gemcitabine	Liposomes	Increase the cytotoxic effects of gemcitabine	[35]
	Camptothecin	Nanosponges of β-cyclodextrin	Increase the overall survival of mice and reduce their growth and metastasis	[36]
	Rsveratrol	Polyethylene glycol and polycaprolactone nanoparticles	Inhibit the growth of docetaxel/doxorubicin-resistant ATC cells	[38]
Differentiation agent delivery	All-trans retinoic acid	Liposomes	Induce differentiation of ATC cells and minimize side effects	[49]
	Tyrosine kinase inhibitors	Extracellular vesicles	Increase the mRNA expression levels of TSHR, NIS, and PAX-8 in ATC cells	[53]
Radioiodine delivery	^131^I	Human serum albumin - MnO_2_	Improve the hypoxic tumor microenvironment and radiotoxicity	[54]
	^131^I	Mesoporous silica nanoparticles	Increase nanoparticle targeting and thus radiotoxicity	[56]
	^131^I	Tyrosine–hyaluronic acid–polyethyleneimine	Increase nanoparticle targeting, reactivate TP53 mutants, and improve radiotoxicity	[59]
Gene delivery	NIS gene	Poly-ethylenimine and PEG	Improve the ability to target ATC cells, increase the expression of NIS, and enhance iodide uptake activity	[63]
	mRNA encoding NIS	Liposome	Increase NIS expression and facilitate ^131^I absorption	[67]
	siRNA targeting BRAF	The near-infrared fluorescent polymer	Suppress tumor cell survival, decrease micrometastases, and facilitate noninvasive NIR imaging	[68]
	siTERT	Poly (D, L-lactide-co-glycolide) modified with chitosan	Reduce tumor growth, migration, and vascularization with no apparent toxicity	[73]
	human telomerase reverse transcriptase promoter	The plasmids wrapped by chitosan nanoparticles	Increase the driving efficacy of the promoter and modulate the expression of double suicide genes	[76]
	microRNA-34b-5p	Liposomes	Inhibit ATC growth via modulation of VEGF-A	[79]
Co-delivery	17-AAG and Torin 2	Mesoporous silica nanoparticles	The low dose of 17-AAG combined with Torin2 could lead to higher cytotoxicity	[81]
	C225 and Au-PFH-NAs	Au-PFH-NAs	Low safety risks and significant synergistic therapy against ATC	[88]
	C225 and 10-HCPT	Polymer (lactic acid-co-glycolic acid)	Achieve precisely targeted diagnosis and synergistic therapy against ATC without obvious system toxicity	[89]
Photothermal therapy	Au	Au nanoparticles	Demonstrate selective and cytotoxic effects on ATC cells	[91]
Combination therapy	^64^Cu and CuS	PEG-CuS nanoparticles	Delay tumor growth, prolong the median survival time, and facilitate noninvasive imaging	[94]
	^131^I and CuS	Bovine serum albumin -CuS nanoparticles	Treat the tumor with lower laser power and temperature, which leads to fewer side effects	[95]
	^131^I and cerebroid polydopamine (CPDA)	CPDA nanoparticles	Show high photothermal conversion efficiency, iodine labeling efficiency, and good stability	[96]
	^131^I and indocyanine green (ICG)	ICG	Increase the cytotoxicity and facilitate noninvasive imaging	[97]
	Bevacizumab and IR825	Polymer (lactic acid-co-glycolic acid)	Achieve synergistic anti-angiogenic therapy/photothermal therapy and multimodal imaging-guided diagnosis	[99]

## Nanomedicine for ATC drug delivery

Besides surgery, various kinds of anticancer agents were employed for ATC therapy. The anticancer drugs, however, had poor therapeutic efficacy due to their poor solubility and permeability, non-targetability, tumor resistance, and systemic toxicity, causing severe side effects. The combination of nanotechnology-based drug delivery systems and multiple anticancer agents ushers in a new era for cancer treatment, significantly improving therapeutic efficacy while causing fewer side effects. Based on the available evidence, nanomaterials have many advantages in the delivery of anticancer drugs. Firstly, they can improve the solubility and stability of the drugs. Secondly, they can prolong the blood circulation time of the drugs and increase their cellular uptake efficacy. Additionally, they improve the targeting ability of cancer cells through the enhanced permeability and retention (EPR) effect or grafting targeting ligands. Nanomaterials provide a promising platform for delivery, including chemotherapeutic drugs, differentiation agents, radioiodine, genes, and co-delivery.

### Nanomedicine for chemotherapeutic drug delivery

One of the main treatment methods in cancer therapy is the application of chemotherapeutic agents. Chemotherapy is widely used in ATC patients and is an independent prognostic factor that is linked to better survival in these patients [21–23]. Unfortunately, it doesn’t seem that patients with ATC respond as well to this strategy. The adverse outcomes of chemotherapy in ATC patients are frequently related to systemic toxicity and chemoresistance, resulting in an average progression-free survival (PFS) of less than three months [4]. The primary mechanisms behind the chemoresistance of ATC are insufficient intracellular drug accumulation caused by ineffective drug absorption and increased drug efflux [24]. Because of their properties of focused drug delivery, enhanced drug absorption, and low adverse effects, nanoparticles (NPs) have shown promising prospects as carriers for chemotherapeutic drug delivery.

Doxorubicin (DOX) induces cell apoptosis by inhibiting the enzyme topoisomerase II and is the only drug approved for the treatment of ATC in monotherapy [25]. Unfortunately, DOX has drawbacks like low effectiveness, significant toxicity, and inefficient treatment. To enhance the drug sensitivity of tumor cells, mesoporous organosilica nanoparticles (MONPs), stabilized by bovine serum albumin (BSA), are designed for DOX delivery [24]. MONPs take advantage of their large surface area, good biocompatibility, and easily modified surface [26, 27]. BSA has the ability to boost the internalization of cancer cells and tumor-specific accumulation [28, 29]. The BSA-DOX-MONPs had a particle size of 368.02 ± 8.90 nm, a zeta potential of -22.11 ± 0.96 mV, and a drug loading efficiency of 47.02%. Xiao Han et al. discovered that BSA-DOX-MONPs boosted the intracellular accumulation of DOX in chemotherapy-resistant ATC cells and reduced their chemoresistance by enhancing drug absorption and decreasing drug efflux. Dopamine-melanin nanoparticles (MNPs) are also synthesized for DOX delivery [30]. The excellent biocompatibility of melanin allows DOX-MNPs to be more effectively internalized by ATC cells than free DOX. However, the DOX-loading capacity of MNPs ranged between 12.64 and 19.43%, restricting their use. Extracorporeal shock waves (ESWs) in combination with nanobubbles (NBs) are also intriguing options for DOX delivery in ATC [31, 32]. ESWs are short-duration focused acoustic waves that can be precisely focused in depth to promote plasmalemma permeabilization [33]. New perfluoropentane-cored glycol chitosan NBs are prepared for DOX delivery, and the resulting nanocarriers were 356.2 ± 15.1 nm in size and 30.4 ± 2.85 mV in zeta potential, with a loading capacity of 4.5%. In the xenograft mouse model that received the combined therapy with DOX-loaded NBs and ESWs, the tumor volume and weight were decreased, and the tumor doubling time was prolonged. Notably, the use of NBs resulted in a low accumulation of doxorubicin in the heart, which reduced the cardiotoxicity of DOX. However, the low loading capacity of NBs is a restriction that just cannot be ignored.

Gemcitabine is another choice for ATC chemotherapy. The novel fluorinated nucleoside analogue gemcitabine showed substantial cytotoxic effect against poorly differentiated human thyroid cancer cell lines in a preclinical study [34]. However, it is quickly metabolized and excreted, resulting in a variety of adverse effects and tumor cell resistance. The liposomes composed of ipalmitoyl-sn-glycero-3-phosphatidylcholine monohydrate (DPPC)/cholesterol (Chol)/N-(carbonyl-methoxipolyethylene glycol-2000)-1,2-distearoyl-sn-glycero-3-phosphoethanolamine (DSPE-mPEG2000) (8:3:1 molar ratio) with a high gemcitabine entrapment efficiency were prepared by Marilena Celano et al [35]. Gemcitabine-loaded liposomes were significantly more cytotoxic to ARO cells than the free drug was (increased cell mortality of 2.4 folds vs. control at 0.3 µM), but the benefit of encapsulation was unclear in the absence of comparison to other cells.

**Fig. 2:**
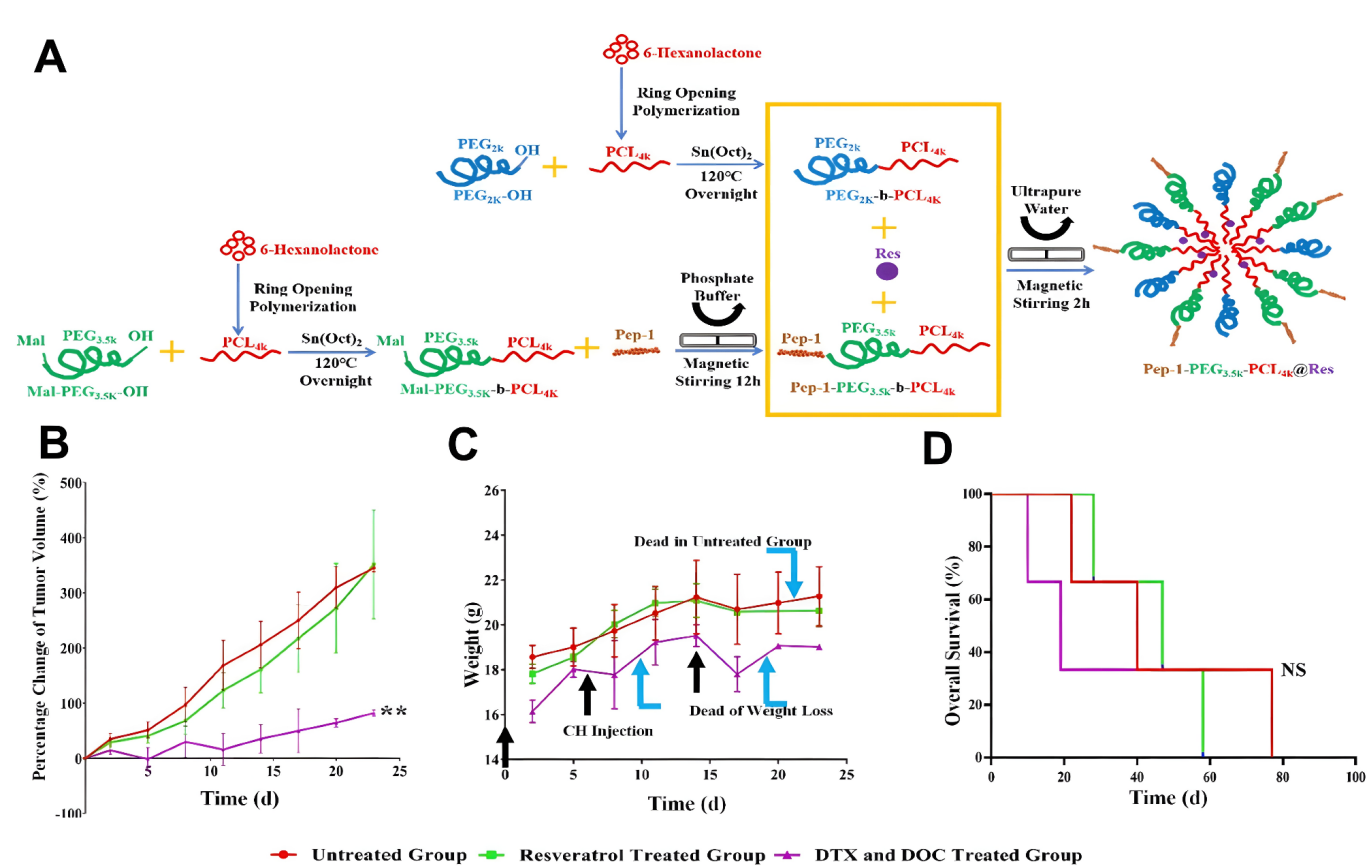
The resveratrol nanoparticles with sustained-release and IL-13Rα2-targeting capacities (Pep-1-PEG_3.5k_-PCL_4k_@Res) were prepared to improve the in vivo resveratrol bioavailability and inhibit the growth of ATC. **A** Schematic illustration of synthesized Pep-1-PEG_3.5k_-PCL_4k_@Res nanoparticles. The change in tumor volume (**B**), body weight (**C**), and overall survival curve (**D**) of the experimental groups. Figure adapted from Le Xiong et al. [38]

The preceding discussions highlighted the role of nanoscale drug delivery systems in enhancing the anti-cancer action of synthetic medicines. It is worth noting that nanoparticles can improve the bioavailability and therapeutic effectiveness of phytochemicals in ATC therapy. Gigliotti et al. designed an β-cyclodextrin-nanosponges encapsulating camptothecin (CN-CPT) [36]. The DNA topoisomerase-I inhibitor CPT, which is derived from the bark of camptotheca acuminata, has anticancer effects [37]. However, it degrades quickly and has low solubility. The CN-CPT nanoparticles, which had a particle size of 350 nm and a zeta potential of -27.4 mV, inhibited the growth of ATC cells by inducing apoptosis and cell cycle arrest. Additionally, CN-CPT significantly increased the overall survival of mice and reduced their growth and metastasis as compared to the control and CPT groups. Resveratrol is also applied to ATC treatment. It is a polyphenolic compound with anti-inflammatory, anti-cancer, and DNA demethylation properties. However, similar to CPT, its high degradation rate limited its bioavailability. Intriguingly, Le Xiong et al. designed interleukin-13 receptor subunit alpha-2 (IL-13Rα2)-targeting and sustained-release nanoparticles to encapsulate resveratrol (Pep-1-PEG_3.5k_-PCL_4k_@Res) [38]. IL-13Rα2 is frequently up-regulated in thyroid cancers, and IL-13Rα2-specific PEP-1 was used to increase the specific binding between NPs and ATC cells [39]. The drug loading and entrapment efficiencies of the Pep-1-PEG_3.5k_-PCL_4k_@Res nanoparticle were 6.81% and 40.84%, respectively. The NPs effectively inhibited the growth of docetaxel/doxorubicin-resistant ATC cells at a rate of 69.23%, providing a potential method to use resveratrol in the clinical treatment of ATCs (Fig. 2).

Overall, these nanoparticles showed good performance in delivering therapeutic drugs to targeted sites and protecting bioactive molecules from inactivation. What we cannot ignore, however, is that the low loading capacity of nanoparticles restricts their widespread use and clinical translation. Furthermore, the research on inorganic nanoparticles in ATC drug delivery is rather limited. Functionalization of inorganic nanoparticles may be a promising method for drug delivery in ATC, and future studies are warranted.

### Nanomedicine for differentiation agent delivery

Although ATC derives from thyroid follicular cells, these cells lose thyroid-like features, such as decreased radioiodine avidity, leading to a very poor prognosis. Radioactive iodine (RAI) is one of the main treatment methods for differentiated thyroid cancer and has the advantages of being safe, efficient, and affordable. However, it is ineffective for the treatment of ATC. Studies showed that reduced expression or deletion of the sodium iodide symporter (NIS) protein resulted in resistance to radioactive iodine therapy in ATC cells [40–42]. It might be a practicable and useful method to increase NIS expression and function to raise RAI sensitivity in ATC patients. Inducing endogenous gene expression by differentiation agents and introducing exogenous genes into ATC cells are two common methods used to increase NIS expression. Most of the details about NIS gene delivery will be outlined in the section on nanomedicine in gene delivery, and the current section focuses on the application of nanomaterials for differentiation agent delivery.

Classic thyroid cancer differentiation agents such as retinoic acids (RA), aromatic fatty acids, histone deacetylase inhibitors, and peroxisome proliferator-activated receptor-gamma agonists (PPAR-γ) induce NIS gene expression [43, 44], but these drugs face challenges due to their poor bioavailability, high dose requirements, and nonspecific targeting [45, 46]. Finding an appropriate method for effective drug delivery is crucial. All-trans retinoic acid (ATRA) causes a considerable remission of several types of cancer due to its property of inducing cell differentiation [47, 48]. The liposomes composed of DPPC/Chol/DSPE-mPEG2000 (6:3:1 molar ratio) were designed for encapsulating ATRA because they can modify the biopharmaceutical properties of the encapsulated molecules and improve therapeutic stability [49, 50]. The PEG-coated liposomes were characterized by a mean diameter of about 200 nm, a polydispersity index of 0.1, and a negative surface charge. The ATRA-loaded liposomes provided the best antitumor activity on FRO cells, and FRO cell viability decreased by up to 80%. Additionally, the PTC-1 cells showed poor sensitivity to the effects of ATRA, indicating that ATRA has a differentiating effect on ATC cells. ATRA-loaded nanostructures showed potential for treating ATC while minimizing side effects. Tyrosine kinase inhibitors (TKIs) are also promising differentiation agents for thyroid cancer. Extracellular vesicles (EVs), which are tiny lipid bilayer-enclosed vesicles released by cells, have emerged as a promising option for drug delivery [51, 52]. Ramya Lakshmi Rajendran et al. found that the TKI delivered by the EVs increased the mRNA expression levels of thyroid stimulating hormone receptor (TSHR), NIS, and paired box-8 (PAX-8) in ATC cells significantly more than the free form of TKI [53]. They then proved that TKI-loaded EVs improve the reinforcement of iodine absorption by virtue of NIS.

When conventional medications fail, the application of differentiation agents is an appropriate and effective strategy to achieve ATC redifferentiation. The therapeutic efficacies of differentiation agents administered by traditional routes such as oral and intravenous were so poor that a new mode of administration was required. Nanomaterials acting as drug carriers play a crucial role in drug delivery. However, inorganic and polymer nanoparticles have received little attention about their potential for redifferentiation agent delivery in ATC, and more studies are required to fully investigate these possibilities.

### Nanomedicine for radioiodine delivery

Traditional radioiodine therapy is ineffective due to the non-iodine-concentrating properties of ATC. Surprisingly, nanoparticles can deliver radioiodine to tumor sites via active or passive targeting, providing an appropriate strategy for ATC therapy.

**Fig. 3:**
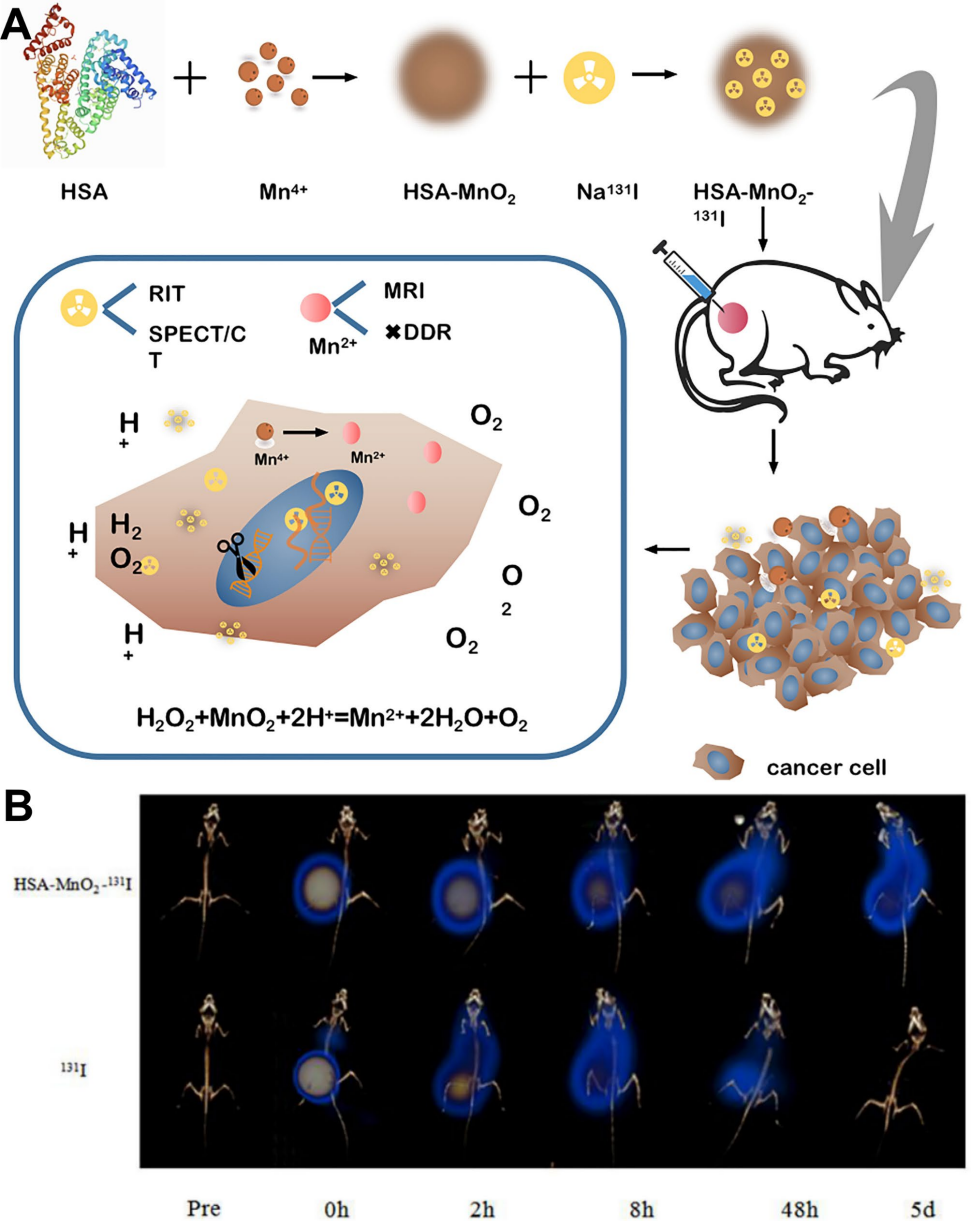
^131^I and MnO_2_ were constructed into a multifunctional NP for SPECT/CT imaging and RIT to improve the tumor hypoxic microenvironment. **A** Schematic illustration of synthesized ^131^I-HSA-MnO_2_ nanoparticles. **B** SPECT/CT imaging of tumors after situation injection of ^131^I-HSA-MnO_2_. Figure adapted from Ziyu Yan et al. [54]

Ziyu Yan et al. proposed a new method using multi-functional nanomaterials to concentrate radiation energy and enhance tumor oxygenation [54]. They synthesized human serum albumin (HSA)-manganese dioxide (MnO_2_) labeled with iodine-131(^131^I). MnO_2_ can enhance the effectiveness of radiotherapy (RT) and improve the hypoxic tumor microenvironment [55]. Western blotting results revealed that the ^131^I-HSA-MnO_2_ therapy dramatically reduced the level of hypoxia inducible factor-1α (HIF-1α), demonstrating the reversal of the hypoxic tumor microenvironment. Furthermore, the ^131^I-HSA-MnO_2_ NPs were significantly aggregated in the tumor compared to other groups, showing improved biocompatibility and negligible biological toxicity (Fig. 3). The ^131^I -HSA-MnO_2_ in particular can be employed for noninvasive imaging, which proved beneficial for the tumor’s diagnosis and therapy. Unfortunately, the NPs’ target ability was relatively poor. Finding an effective target to deliver radioiodine to the tumor site can improve the targeting ability and therapeutic effects. Vascular endothelial growth factor receptor (VEGFR) is highly expressed in ATC cells and is a suitable therapeutic target for ATC treatment. Ruiguo Zhang et al. designed ^131^I-labeled anti-VEGFR2 targeted mesoporous silica nanoparticles (^131^I-BSA-MSNs-anti-VEGFR2), achieving the co-delivery of anti-VEGFR2 and ^131^I [56]. The concentration in the tumor of the ^131^I-BSA-MSNs-anti-VEGFR2 group at 24 and 72 h post-injection was 32.2 ± 2.8%ID/g and 23.0 ± 1.8%ID/g, respectively, when compared to the non-targeted group (26.1 ± 2.5%ID/g and 12.3 ± 1.2%ID/g, respectively). The ^131^I-BSA-MSNs-anti-VEGFR2 nanoparticles increased targeting and thus radiotoxicity, showing efficiency at preventing tumor growth and extending median survival. Cluster determinant 44 (CD44) overexpression and TP53 frequent mutations have been observed in many types of ATC cell lines, and cancer cells harboring TP53 mutations were characterized by radiation ionizing insensitivity [57–59]. Tyrosine (Tyr)-hyaluronic acid (HA)-polyethyleneimine (PEI) was designed as a CD44-targeted delivery system loading Prima-1 and ^131^I [59, 60]. Prima-1 is a mutant-TP53 restoring agent that might be used to reduce radiation resistance in ATC. The CD44-targeted agent increased nanoparticle targeting and effectively delivered ^131^I and Prima-1 to the tumor site through nanocarriers. Studies conducted *in both vitro* and in vivo showed that Prima-1 sensitized ATC cells with TP53 mutations in reaction to ^131^I internal radiation therapy by functionally reactivating the mutants, demonstrating excellent therapeutic synergy.

The nanoparticles made it possible to apply radioiodine for ATC treatment. MnO_2_-based nanocomposites were used for enhanced RT and acted as nanocarriers to deliver radioiodine to tumor sites via passive targeting. However, the passive targeting ability of nanoparticles was relatively poor, resulting in possible radiation harm to normal tissue. Nanocarriers labeled by targeting ligands can increase nanoparticle targeting and radioiodine accumulation in the tumor site through active targeting. Therefore, combining radiosensitizers and targeting ligands with nanocarriers may be an encouraging strategy for maximizing the efficiency of radioiodine therapy. MSNs and polymer-based nanoparticles have been shown to be excellent nanocarriers for ATC radioiodine therapy, and other nanomaterials, such as metal- and lipid-based nanoparticles, may be promising candidates.

### Nanomedicine for gene delivery

In recent years, much attention has been directed toward the application of gene therapy for cancer, and various kinds of gene therapy approaches have been applied for cancer treatment. However, the low accumulation of gene medications within tumor tissues and their rapid degradation preclude their widespread use. By improving the in vivo delivery of gene therapeutic agents to solid tumor tissues through the EPR effect, nanotechnology has significantly facilitated the clinical translation of nucleic acid drugs for cancer therapy [61, 62].

**Fig. 4:**
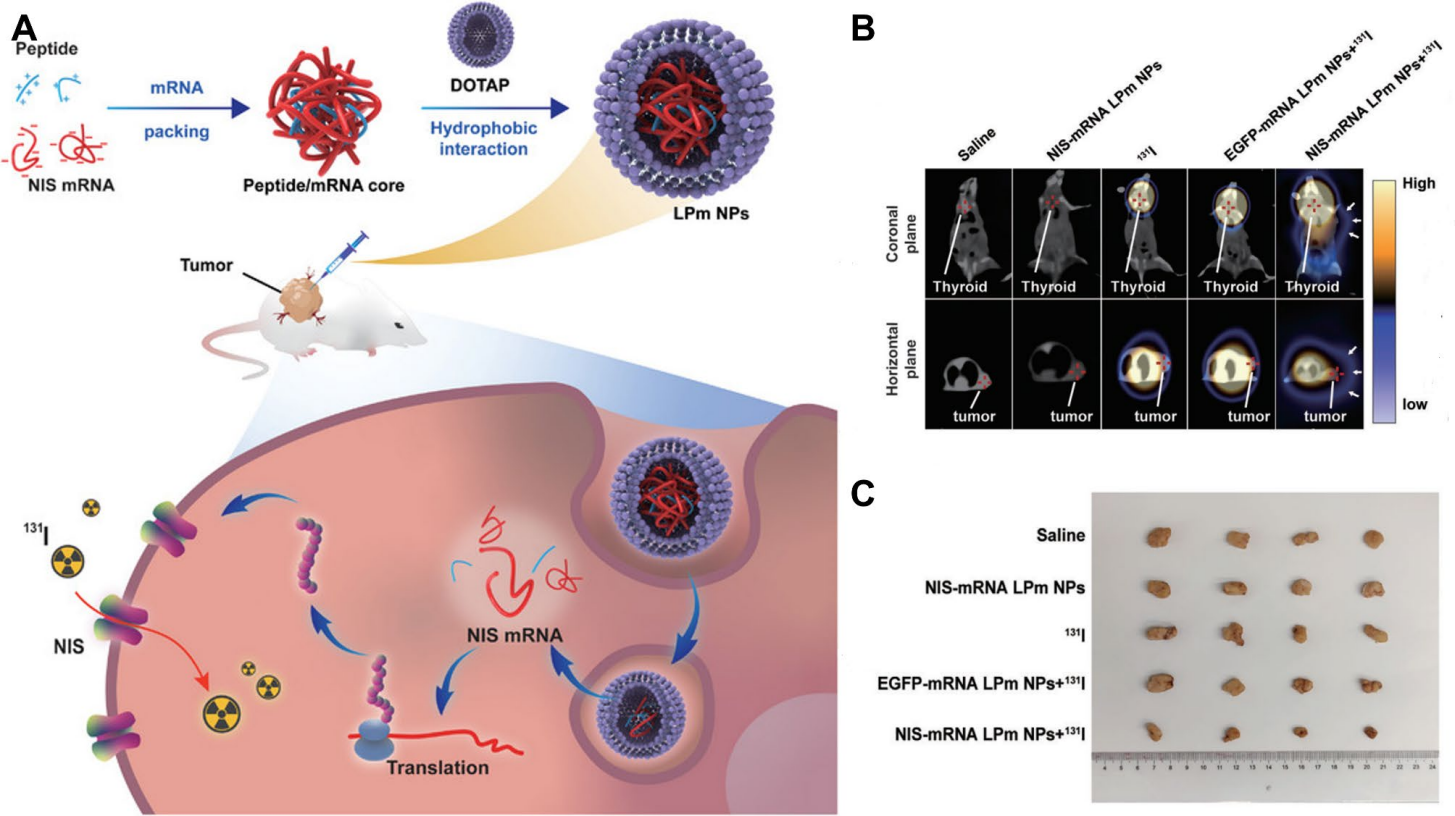
LPm NP exhibited favorable performance in delivering NIS mRNA and ^131^I, demonstrating promising adjunctive therapy for patients with ATC by restoring iodine affinity and enhancing the therapeutic efficacy of radioactive iodine. **A** Schematic diagram of the structure of LPm NPs and their function of transporting NIS mRNA in cells for protein expression. **B** SPECT/CT imaging of mice treated with saline, NIS-mRNA LPm NPs, ^131^I, EGFP‐mRNA LPm NPs + ^131^I, or NIS‐mRNA LPm NPs + ^131^I for 24 h. **C** Image of the excised subcutaneous tumor on day 18. Figure adapted from Qinglin Li et al. [67]

As mentioned previously, gene delivery is used to increase NIS expression. Selective NIS gene transfer into tumor cells has been developed for decades, but the poor biostability and low delivery effectiveness of ‘’naked’’ nucleic acids have hampered their use. To get around these, GE11 was coupled to linear poly-ethylenimine (LPEI) via a PEG link (LPEI-PEG-GE11), which was designed for the delivery of the NIS gene [63]. GE11 is a specific ligand for the endothelial growth factor receptor (EGFR). ATC cells, which contain an overexpressed version of the EGFR, are more easily targeted by the GE11-modified synthetic polymers. Iodide uptake of the cells was noticeably higher following transfection with targeted LPEI-PEG-GE11/NIS compared to other groups, indicating better transfection effectiveness with the peptide ligand GE11. Furthermore, the ability of EGFR-targeted nanoparticles to target NIS to ATC was demonstrated in vivo by xenograft mice treated with an application of LPEI-PEG-GE11/NIS and ^131^I. Compared to control groups, these mice showed a significant delay in tumor growth and an increase in survival time. Chemically modified mRNA, which has a higher transfection effectiveness than DNA, has drawn a lot of attention in tumor therapy [64]. However, it is difficult to transport across the cell membrane barrier and is readily degraded in the absence of a delivery system [65]. With advances in nanotechnological methods in pharmaceuticals, a range of nanocarriers for in vivo delivery of mRNA have been developed [66]. To increase the expression of NIS and the sensitivity of ATC to radioiodine treatment, Qinglin Li et al. synthesized lipid-peptide-mRNA nanoparticles (NIS-mRNA LPm NPs) to transport mRNA encoding NIS to ATC [67]. *In vitro* experiments showed that the NIS-mRNA LPm NPs group increased NIS expression in 8305C cells by more than 70-fold when compared to the negative control and naked NIS-mRNA, and only NIS-mRNA LPm NPs could increase cellular iodine uptake. Additionally, intratumoral injection of NIS-mRNA LPm NPs induced local NIS expression and promoted ^131^I absorption more than 4000 times compared to other ^131^I-treated groups (Fig. 4).

Nanoparticle-mediated delivery of RNA interference (RNAi) agents to solid tumors for the specific silencing of target genes driving development and/or metastasis is also an effective method for cancer therapy [61]. Yanlan Liu et al. designed the near-infrared (NIR) fluorescent polymers that the poly[2,6-(4,4-bis-(2-ethylhexyl)-4H-cyclopenta[2,1-b;3,4-b′]dithiophene)-alt-4,7(2,1,3-benzothiadiazole)] (polyPCPDTBT) selected as the building block, with a surface coating of 1,2-distearoyl-*sn*-glycero-3-phosphoethanolamine-*N*-[methoxy(polyethylene glycol)]. Due to the NIR fluorescent polymers’ small size, lengthy blood circulation, and significant tumor site accumulation, the delivery of short-interfering RNA (siRNA) targeting serine/threonine-protein kinase B-Raf (BRAF) to the ATC site by NIR polymeric NPs enables the inhibition of tumor growth and metastases [68]. BRAF activation and upregulation in ATC patients result in cellular proliferation, invasion, metastasis, and dedifferentiation [69]. By increasing the internalization of siRNA in ATC cells, the nanoparticles reduced BRAF expression to suppress tumor cell survival and decrease micrometastases. Compared with NP (siControl), NP (siBRAF) showed a 60% reduction in BRAF protein expression and a threefold reduction in tumor size. Furthermore, there were considerably fewer micrometastases in NP (siBRAF)-treated mice than in NP (siControl) mice. Notably, after systemic administration of siRNA NPs, the NIR fluorescent polymer can be used for noninvasive NIR imaging to track the kinetics of whole-body biodistribution and tumor localization. Another suitable molecular target for the treatment of ATC is telomerase reverse transcriptase (TERT). The most common event in ATC patients has been found to be TERT gene alterations, which have been suggested as the best target for ATC therapy [70–72]. Biodegradable poly (lactic-coglycolic acid) (PLGA) nanoparticles were modified with chitosan to provide their complexation with an anti- human telomerase reverse transcriptase (hTERT) oligonucleotide [73]. The anti-hTERT nanoparticles (Na-siTERT) caused a significant 40% decrease in cell viability and migration when compared to free siTERT. Additionally, it was discovered that intravenous administration of Na-siTERT significantly reduced the growth, ability to invade, and vascularization of the xenograft mice, with no obvious indications of toxicity.

The suicide gene/prodrug system is another strategy for gene therapy, modulating expression of a suicide gene with the promoters mainly destroying tumor cells while leaving the surrounding tissues unharmed. It was discovered that double suicide genes driven by the human telomerase reverse transcriptase promoter (hTERTp) can be specifically programmed in ATC for controllable treatment [74]. However, the weak activity of hTERTp limits its application [75]. Aoshuang Chang et al. designed novel chitosan-based nanoparticles containing genes that hTERTp added a radiation enhancer (E9) to and were labeled by ^131^I [76]. The use of the radiation enhancer E9 increased the driving efficacy of the promoter under the weak radiation of ^131^I. Compared to the control groups, cancer cells treated with the constructed nanoparticles exhibited a significant decrease in cell viability and an increase in cell apoptosis (P < 0.05).

Targeting noncoding RNAs (ncRNAs) is also used in gene therapy. The best-known ncRNAs are the microRNAs (miRs), whose abnormal expression is linked to the development of malignancy [77]. The microRNA-34b-5p (miR-34b) had a suppressive effect on ATC via modulation of VEGF-A [78]. Hydration-of-freeze-dried-matrix (HFDM) formulated liposomes were used for effective delivery of miR-34b to ATC [79]. The miR-34b NPs had a particle size of 135.3 ± 10.80 nm, a zeta potential of 39.16 ± 0.451 mV, and a drug entrapment efficiency of 96.9 ± 2.18%. MiR-34b expression was low in ATC cells, and a significant overexpression (p < 0.05) was observed after transfection with liposome-loaded miR-34b. Additionally, in vivo study found that administering miR-34b-loaded liposomes intravenously significantly decreased the volume of the tumors (7 ± 1.2 mm^3^) when compared with those of miR-1-loaded liposome (16 ± 0.9 mm^3^) and the empty liposome (18 ± 0.6 mm^3^) groups at the end of this study (p < 0.05), showing excellent antitumor effects by significantly decreasing VEGF-A expression.

Although gene therapy has opened a new window in ATC therapy, its efficacy in preclinical and clinical trials appears to be limited due to low accumulation and rapid degradation. Recently, as the approach to systematic cancer treatment converts toward individualized therapy, personalized gene therapy has played a prominent role in treating ATC with NPs delivering nucleic acids to targeted tumor sites. Based on the above studies, lipid- and polymer-based nanomaterials have been shown to be excellent nanocarriers for gene delivery, while inorganic nanomaterials have been largely ignored. Compared to traditional lipid- and polymer-based nanocarriers, inorganic nanomaterials are emerging as potential gene delivery vehicles due to their advantages of tunable size, storage stability, excellent pharmacokinetic profiles, and imaging capability. And further studies should be performed to explore more possibilities.

### Nanomedicine for co-delivery

The current therapies for ATC patients are not sufficient because many challenges exist, such as their no-differentiation and aggressive nature, complex tumor microenvironment, and intrinsic resistance to drugs. Drug-combined therapies have shown increasing benefits against those challenges. The nanocarriers have been developed for combination therapy to overcome the inherent limitations of monotherapy and reduce tumor recurrence and metastasis, providing possible approaches to overcome therapeutic challenges. This section focuses on the possible application of nanomaterials for co-delivery in ATC therapy.

Congcong Wang et al. constructed a new 2-in-1 MSNs targeting VEGFR2 ((17-AAG + Torin2) @MSNs-anti-VEGFR2 ab), containing 17-allylamino-17-demethoxygeldanamycin (17‑AAG) and 9‑(6‑Aminopyridin‑3‑yl) ‑1‑(3‑(trifluoromethyl)phenyl) benzo[h] [1,6] naphthyridin-2(1H)-one (Torin2) [80]. 17‑AAG, a novel inhibitor to act on heat shock protein 90 (HSP90), has shown great promise with significant biological activity [81]. Excessive activation of the mammalian target of rapamycin (mTOR) has been revealed in ATC and Torin2 inhibits cell growth, survival, proliferation, and migration in ATC due to the dual inhibition effect of mTORC1 and mTORC2 [82, 83]. *In vitro* studies found that synergistic effects occurred in FRO cells when the concentration ratios of 17-AAG and Torin2 were 1:1 or 2:1, and the median inhibition concentration (IC_50_) of FRO cells was 0.33 and 0.26 µM, respectively. In vivo treatment showed that tumors treated with the (17‑AAG + Torin2) @MSNs-anti-VEGFR2 group had degeneration and massive necrosis of tumor cells, and cell proliferation and angiogenesis were evidently decreased. Furthermore, the median survival time of the group treated with (17-AAG + Torin2) @MSNs-Anti-VEGFR2 ab was 27.5 days, which was significantly prolonged compared with the group treated with (17-AAG + Torin2) @MSNs or normal saline (all P < 0.01).

**Fig. 5:**
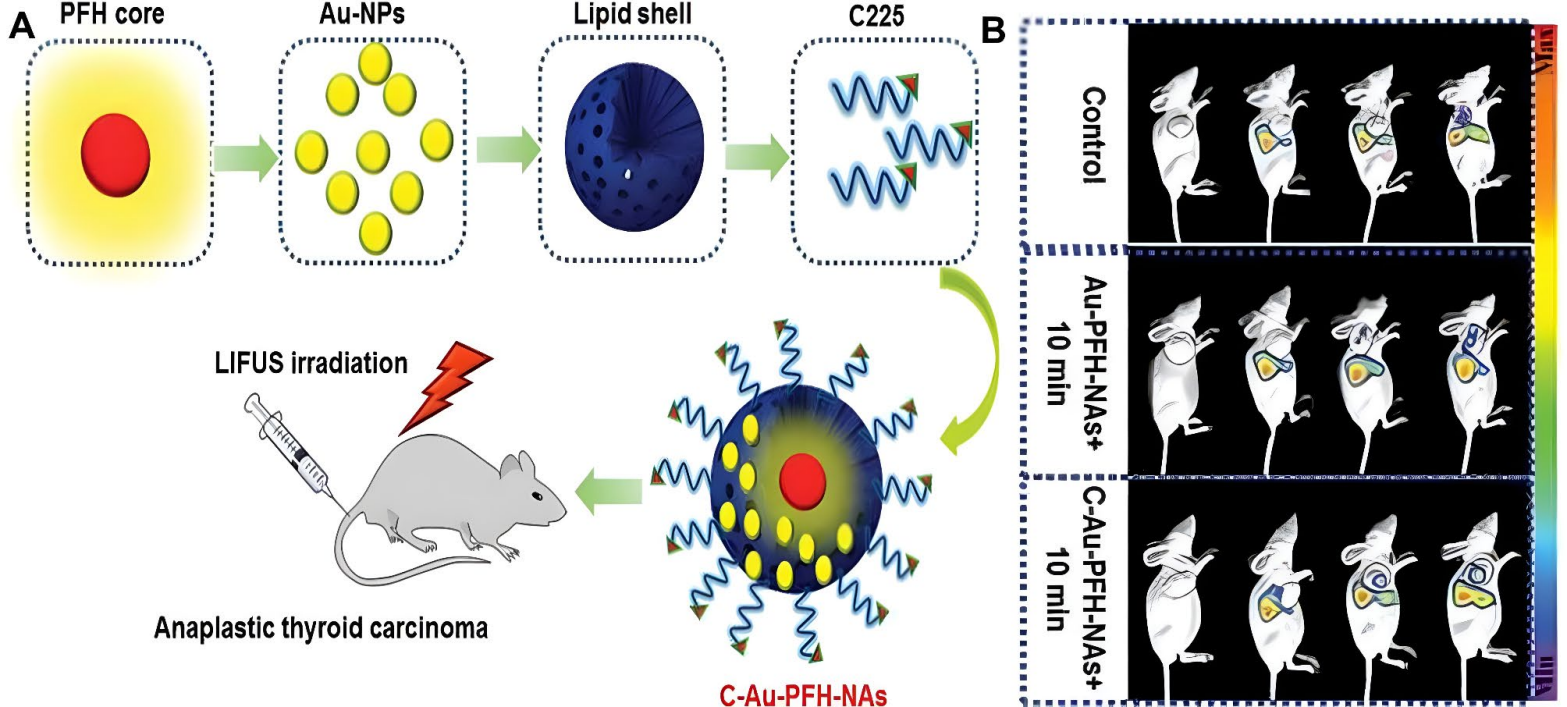
C-Au-PFH-NAs were designed for low-intensity focused ultrasound diagnosis ablation of ATC. **A** Schematic illustration of synthesized C-Au-PFH-NAs. **B** Ultrasound imaging of tumors after situation injection of C-Au-PFH-NAs. Figure adapted from Ying Liu et al. [88]

Cetuximab (C225) is an EGFR-targeted monoclonal antibody which is also used for ATC treatment. Because of its noninvasive nature and significant tissue-penetrating ability, low-intensity focused ultrasound (LIFU) has been extensively studied for tumor therapy and imaging detection [84–86]. Couple triggerable drug-charged nanocarriers with LIFU has been proven to enable controlled drug release for personalized treatment [87]. The conjugation of C225 to gold-perfluorohexane nanoparticles (C-Au-PFH-NAs) is a novel method [88]. Au-PFH-NAs have similar effects to CPT-11, which is a derivative of camptothecin. LIFU-triggered C-Au-PFH-NAs showed higher accumulation efficiency of nanoparticles at tumor sites and boosted cytotoxicity by increasing cell membrane permeability, showing significant synergetic antitumor effects (Fig. 5). Yang Wang et al. also synthesized EGFR-targeted phase-changeable polymer nanoparticles (C-HPNs) loaded with C225 and 10-hydroxycamptothecin (10-HCPT), which could be triggered by LIFU [89]. 10-HCPT is a kind of camptothecin analog. Not only can the combination therapy of C225 and 10-HCPT afford nanocarrier targeting ability, but it also shows potent antitumor effects. Combining C-HPNs and LIFU had a remarkable therapeutic impact in vivo, inhibiting tumor growth in nude mice without producing severe side effects. Significantly, not only can LIFU facilitate chemotherapeutic drug release on demand, but it also enhances ultrasound imaging by LIFU-induced acoustic droplet vaporization, achieving precise diagnosis and synergistic therapy against ATC simultaneity.

ATC is such an aggressive tumor that monotherapy cannot completely eradicate it, which paves the way for combination therapy. Serving as a link between different treatment modalities, the nanoparticles play an indispensable role in ATC combination therapy. Nanotechnology was applied to construct nanoparticles for co-delivery, achieving drug delivery with lower systemic toxicity and better targeting. However, the combination of targeted therapy and other types of therapeutic modalities is still in its early stages, and their synergistic mechanisms still need further study. Furthermore, more possible combinations should be further explored.

## Nanomedicine for direct ATC therapy

Nanocarrier-based drug delivery systems have shown tremendous potential for cancer therapy. Notably, under exogenous stimulation, nanomedicine can act as a flexible platform for additional therapeutic modalities, such as photothermal therapy (PTT), to efficiently treat tumors. Photosensitizer-based PTT therapy has drawn extensive attention as a relatively moderate and noninvasive alternative for ATC treatment. PTT utilizes the photothermal effect of photothermal transduction agents (PTAs) to harness light energy and convert it into heat, thereby elevating the local temperature and inducing cancer cell death [90]. The PTAs have excellent tumor accumulation, high photothermal conversion efficiency (PCE), and the ability to combine various imaging modalities and therapeutic activities into a single platform. Furthermore, PTT can be combined with the current available therapies and present promising synergistic therapeutic efficiency.

### Nanomedicine for photothermal therapy

With the benefits of being easy to implement, non-invasive, and having minimal side effects, PTT is a newly developed and encouraging therapeutic strategy. The current section highlights the application of PTT in the treatment of ATC.

Gold nanoparticles (AuNPs) are the most commonly used metal nanoparticles due to their biocompatibility and their unique physicochemical properties, such as high surface area, small size, chemical stability, and versatile optical properties, which can be activated when irradiated by NIR light and convert the light into heat, resulting in cell death. Additionally, AuNPs can be functionalized with different moieties depending on their use. Mariana Amaral et al. designed a gold-based nanoplatform in which AuNPs were coated with hyaluronic acid and oleic acid (HAOA) and functionalized with endothelial growth factor (EGF) or lapatinib to target endothelial growth factor receptor (EGFR), or holo-transferrin (HTf) to target type 1 transferrin receptor (TfR1/CD71) [91]. Based on their physicochemical properties, such as average size distribution, conjugation efficiency, absorbance spectra, and morphology, HTf was proven to be the most appropriate ligand. Furthermore, the HTf-functionalized HAOA-coated AuNPs exhibited the highest cytotoxicity against the ATC cell line, resulting in a decrease in cell viability of 22%, making it the most effective formulation. Notably, it has been shown to be secure and biocompatible.

PTT has the advantages of strong efficacy and minimal invasion, and AuNPs as a PTA assume a central role. AuNPs have a high light-to-heat conversion efficiency and good thermal stability, as well as the capacity to spontaneously reach the tumor site and accumulate in the malignant vascularized area, making them a perfect ally for PTT. However, the main limitations are the possible AuNP toxicity and their accumulation in off-target organs, and more studies are needed to explore how to minimize the toxicities brought by AuNPs.

### Nanomedicine for combination therapy

Monotherapy is insufficient for ATC therapy, and PTT is not an exception. Synergistic effects can be achieved through the integration of different therapies, making combination therapy a promising approach. PTT has the ability to modulate tumor perfusion and stimulate vascular permeability, rendering it a favorable option for the combination of other therapies that aim to achieve synergistic effects and improve treatment outcomes. This section highlights the combination of PTT with internal isotope therapy and targeted therapy.

Metal nanoparticles combined with radionuclides enhanced the therapeutic effect through radio-sensitization and synergistic effects. Copper sulfide (CuS) is a photothermal reagent and has been used for photothermal therapy (PTT) in several studies [92, 93]. Copper-64 (^64^Cu) can be used for RT and combined with PPT for the treatment of ATC. Min Zhou et al. designed PEG-coated [^64^Cu] CuS NPs (PEG-[^64^Cu] CuS NPs) [94]. The combination of radiotherapy and photothermal therapy demonstrated a significantly superior effect in delaying tumor growth and prolonging the median survival time compared to PTT alone. Furthermore, ^64^Cu is a suitable radioisotope for positron emission tomography (PET) imaging and was used to evaluate treatment efforts. Another promising option for ATC therapy is radioiodine. Chunmei Zhang et al. synthesized ^131^I-labeled BSA-modified CuS nanoparticles (^131^I-BSA@CuS) with attributes of both RT and PTT for the treatment of ATC [95]. The BSA@CuS nanoparticles exhibited a conversion efficiency of 28.07% in rapidly and effectively converting the light energy from an 808 nm laser into thermal energy. The tumors shrank in the observation period after ^131^I-BSA@CuS treatment, whereas no such changes were observed in the other groups, showing excellent therapy effects. The study also demonstrated that combination therapy resulted in lower laser power and temperature requirements for effective tumor treatment compared to single PTT, potentially reducing the incidence of side effects.

**Fig. 6:**
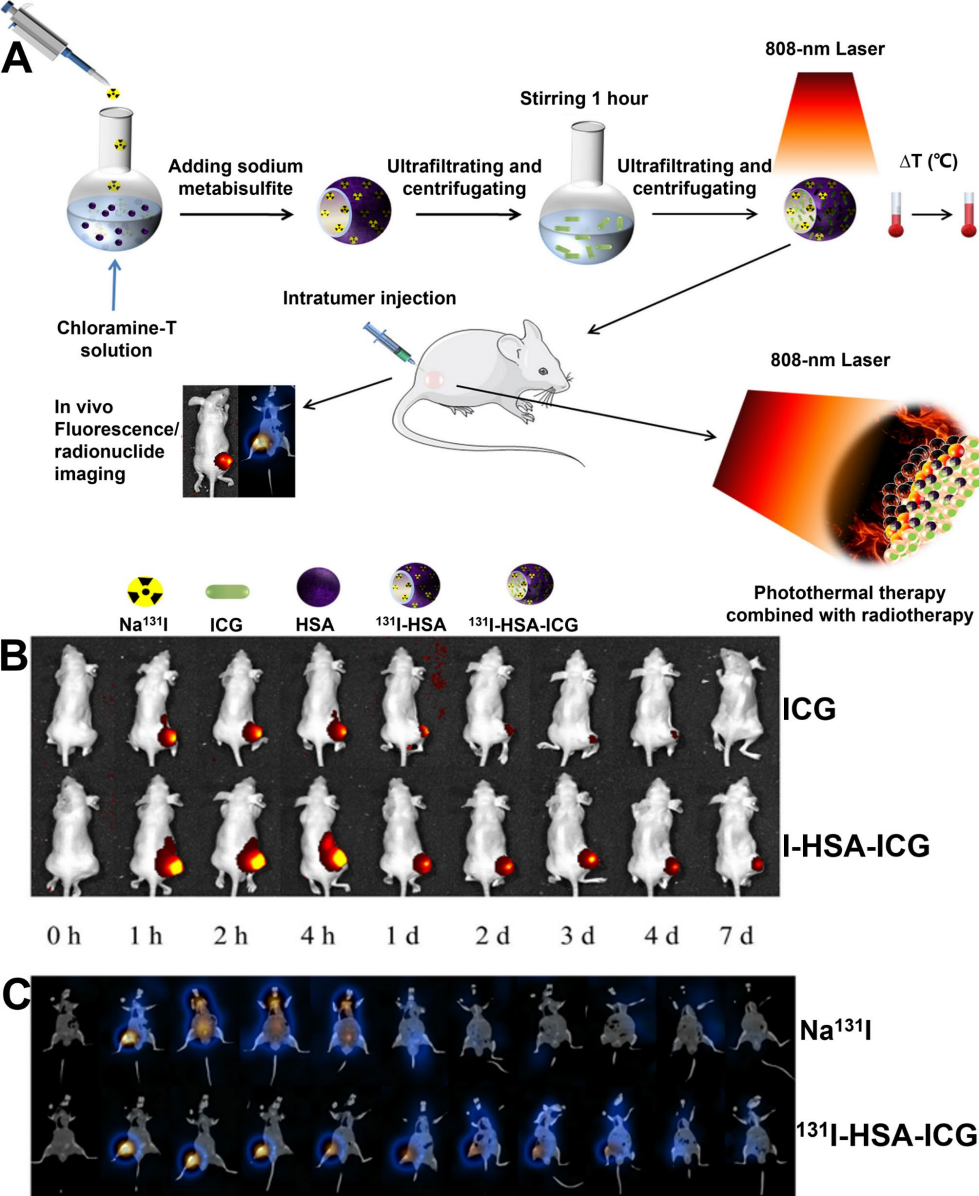
^131^I and ICG were constructed into a multifunctional NP for locally combined photothermal and radionuclide therapy on iodine-refractory thyroid cancer and ATC. **A** Schematic illustration of synthesized ^131^I-HSA-ICG nanoparticles. **B** In vivo fluorescence imaging of pure ICG and I-HSA-ICG at different time points. **C** SPECT/CT tomographic images of Na^131^I and ^131^I-HSA-ICG at different time points. Figure adapted from Xuemei Zhang et al. [97]

^131^I-radiolabeled cerebroid polydopamine nanoparticles (CPDA-^131^I) were also designed by Shuo Huang et al. to improve the therapeutic effect of ATC [96]. CPDA with a cerebriform morphology and high specific area demonstrated the highest iodine-labeling rate (88.39 ± 5.17%) compared to other types of polydopamine (PDA) nanoparticles, including smooth polydopamine (SPDA) nanoparticles, polyporous polydopamine (PPDA) nanoparticles, macroporous polydopamine (MPDA) nanoparticles, etc. The efficiency of PTT in ATC ablation depends on the conversion efficiency of synthesized nanomaterials, and CPDA-^131^I performed well with a photothermal conversion efficiency of 50.3%. The cellular uptake rate of CPDA-^131^I was also excellent (8.90 ± 0.89%), and it was roughly 130 times greater than that of free ^131^I (0.07 ± 0.00%). In subsequent experiments, the researchers discovered that the CPDA-^131^I group significantly inhibited tumor growth and had a more pronounced synergistic therapeutic effect on 8305C tumors than PTT or RT alone. Another study reported HSA-indocyanine green (ICG) labeled ^131^I for the treatment of ATC [97]. ^131^I-HSA-ICG NPs strongly absorb light at 808 nm and could be heated to over 50°C at non-toxic concentrations. Its photothermal conversion efficiency was 24.25%. The tumor volume was significantly reduced on day 5 after ^131^I-HSA-ICG treatment, with little apparent growth thereafter, showing the most significant ablation effect on tumor cells compared to other groups. Furthermore, ICG could be excited by NIR light to generate strong fluorescence for imaging (Fig. 6).

The combination of targeted therapy and PTT showed some encouraging results in ATC therapy, and the benefits have been confirmed in both *in vitro* and in vivo studies [98]. Qimeihui Wang et al. engineered Bevacizumab (Bev)-modified PLGA NPs loaded with IR825 and perfluoropentane (IR825@Bev-PLGA-PFP NPs, which are termed IR825@B-PPNs) for targeted therapy and PTT of ATC using NIR irradiation. IR825 as a PTA has the advantage of desirable biocompatibility and biodegradability [99, 100]. Bevacizumab is an FDA-approved anti-VEGF monoclonal antibody that possesses a distinct affinity for VEGF and has the advantages of being anti-angiogenesis and ATC-targeted [101]. IR825@ B-PPNs intensively accumulated around the tumor cells, and the tumor was completely eradicated after 18 days with no recurrence after 30 days of constant observation, demonstrating outstanding synergistic antitumor effects. Furthermore, encapsulated PFP and IR825 were employed as imaging agents for ultrasound/photoacoustic/fluorescence multimodal imaging, providing precise diagnosis, real-time monitoring, and guidance.

Although PTT has the advantages of being highly efficacious and non-invasive, its monotherapy still cannot completely eradicate tumors due to non-negligible drawbacks such as local tumor inflammation and immune antigenic lesions. Interestingly, the combination of PTT with other therapies holds great potential for achieving a synergistic effect and enhancing treatment efficacy in ATC. With its many advantages, combination therapy is not without its drawbacks. One serious flaw is the neglect of the investigation of some PTAs for ATC, such as carbon-based nanomaterials, conjugated polymers, and polyaniline nanomaterials. Additionally, PTAs are suitable imaging materials for multiple imaging modalities, and their imaging capability and ability to precisely visualize tumors require further investigation and implementation.

## Challenges and opportunities

There have been many journal publications reporting on innovative nanomedicine-based therapeutic methods for ATC therapy, and good therapeutic effects for ATC have been confirmed in preclinical studies. However, the clinical development of nanomedicines for ATC therapy is still in its early stages. And several ongoing challenges and problems may hamper further clinical applications of the nanomedicines for ATC.

One of the major challenges lies in the fact that predicting human efficacy from animal studies is constrained by the inherent limitations of models, resulting in difficulties with clinical translation. In this regard, developing more diversified and accurate ATC animal models has the potential to significantly improve the clinical translation of nanomaterials, which is critical in advanced nanotherapeutic techniques for ATC. The limitations of nanomaterials themselves in biomedical applications, such as their biodistribution, potential toxicity, and liver/renal clearance of nanoparticles also present significant challenges, which are common to the clinical translation of most nanomedicines [102, 103]. Therefore, more attention needs to be paid to optimizing preparation methods, ensuring biosafety and improving effectiveness of nanomedicine. In addition to the above, the utilization of metal- and carbon-based nanocarriers for drug delivery in ATC remains unexplored, while alternative therapeutic approaches such as photodynamic therapy have been overlooked. Therefore, further research is urgently needed to advance ATC treatment options.

## Conclusion

Although many therapeutic methods, such as surgery, chemotherapy, and radiotherapy, have been tried against cancer with different degrees of success, treating ATC remains challenging for physicians. Nanomedicine has opened a new door to overcome obstacles associated with conventional ATC treatment modalities. Numerous studies have reported that nanotherapeutic approaches have significantly improved the outcome of ATC compared to conventional treatments [104, 105]. By serving as drug carriers, nanomaterials have a surprising potential to enhance the effectiveness of cancer therapeutics. Not only can they deliver a wide range of therapeutic agents to tumor sites via active or passive targeting, but they also greatly improve the internalization of drugs in ATC cells, reducing the survival of cancer cells. Some nanomaterials, such as PTA, are the specialized ones that serve as therapeutic molecules, showing cytotoxicity for ATC cells under NIR irradiation. Additionally, nanostructures can provide platforms for the coexistence of various therapeutic methods, showing unexpected synergistic therapeutic effects in ATC. The nanomedicines have changed the outlook of chemotherapy, differentiation therapy, radioiodine therapy, gene therapy and targeted therapy, and in terms of their required dose, therapeutic efficacy, toxicity, stability, and so on. Furthermore, the nanomaterials provide novel therapeutic methods of ATC, such as PTT. Although there are currently no commercially available nano-based ATC therapeutics and their clinical efficacy remains uncertain, preclinical studies suggest that nanomaterials hold significant potential for enhancing the performance of ATC therapeutics. Therefore, it is critically necessary for diverse areas to work together to speed up the preclinical studies and clinical translation of ATC nanomedicines.

## Data Availability

Not applicable, please refer to the original references.

## Abbreviations

ATC: Anaplastic thyroid cancer
EBRT: External beam radiation therapy
MDR: Multiple drug resistance
EPR: Enhanced permeability and retention
PFS: Progression-free survival
NPs: Nanoparticles
DOX: Doxorubicin
MONPs: Mesoporous organosilica nanoparticles
BSA: Bovine serum albumin
MNPs: Dopamine-melanin nanoparticles
ESWs: Extracorporeal shock waves
NBs: Nanobubbles
DPPC: Ipalmitoyl-sn-glycero-3-phosphatidylcholine monohydrate
Chol: Cholesterol
DSPE-mPEG2000: N-(carbonyl-methoxipolyethylene glycol-2000)-1,2-distearoyl-sn-glycero-3-phosphoethanolamine
CN-CPT: β-cyclodextrin-nanosponges encapsulating camptothecin
IL-13Rα2: Interleukin-13 receptor subunit alpha-2
RAI: Radioactive iodine
NIS: Sodium iodide symporter
RA: Retinoic acids
PPAR-γ: Peroxisome proliferator-activated receptor-gamma
ATRA: All-trans retinoic acid
TKIs: Tyrosine kinase inhibitors
EVs: Extracellular vesicles
TSHR: Thyroid stimulating hormone receptor
PAX-8: Paired box-8
HSA: Human serum albumin
MnO_2_: Manganese dioxide
^131^I: Iodine-131
RT: Radiotherapy
HIF-1α: Hypoxia inducible factor-1α
VEGFR: Vascular endothelial growth factor receptor
MSNs: Mesoporous silica nanoparticles
CD44: Cluster determinant 44
TP53: Tumor protion53
Tyr: Tyrosine
HA: Hyaluronic acid
PEI: Polyethyleneimine
LPEI: Linear poly-ethylenimine
EGFR: Endothelial growth factor receptor
RNAi: RNA interference
NIR: Near-infrared
polyPCPDTBT: poly[2,6-(4,4-bis-(2-ethylhexyl)-4 H-cyclopenta[2,1-b;3,4-b′]dithiophene)-alt-4,7(2,1,3-benzothiadiazole)
BRAF: B-Raf
siRNA: Short-interfering RNA
TERT: Telomerase reverse transcriptase
PLGA: Poly (lactic-coglycolic acid)
hTERT: Human telomerase reverse transcriptase
hTERTp: Human telomerase reverse transcriptase promoter
ncRNAs: Noncoding RNAs
miRs: MicroRNAs
miR-34b: MicroRNA-34b-5p
HFDM: Hydration-of-freeze-dried-matrix
17‑AAG: 17-allylamino-17-demethoxygeldanamycin
Torin2: 9‑(6‑Aminopyridin‑3‑yl) ‑1‑(3‑(trifluoromethyl)phenyl) benzo[h] [1,6] naphthyridin-2(1 H)-one
HSP90: Heat shock protein 90
mTOR: Mammalian target of rapamycin
IC_50_: Median inhibition concentration
C225: Cetuximab
LIFU: Low-intensity focused ultrasound
C-Au-PFH-NAs: Gold-perfluorohexane nanoparticles
10-HCPT: 10-hydroxycamptothecin
PTT: Photothermal therapy
PTAs: Photothermal transduction agents
PCE: Photothermal conversion efficiency
AuNPs: Gold nanoparticles
HAOA: Hyaluronic acid and oleic acid
EGF: Endothelial growth factor
HTf: Holo-transferrin
TfR1/CD71: Type 1 transferrin receptor
CuS: Copper sulfide
^64^Cu: Copper-64
PET: Positron emission tomography
CPDA: Cerebroid polydopamine
SPDA: Smooth polydopamine
PPDA: Polyporous polydopamine
MPDA: Microporous polydopamine
ICG: Indocyanine green
Bev: Bevacizumab
PFP: Perfluoropentane
